# Supplementary material for: Plasma proteome dynamics of COVID-19 severity learnt by a graph convolutional network of multi-scale topology
Source: Life Sci Alliance. 2023 Feb 20;6(5):e202201624. doi: 10.26508/lsa.202201624 (PMC9941303; doi:10.26508/lsa.202201624)
Supplement: Supplementary file 9 [file LSA-2022-01624_TableS1.pdf]

Genome Research draft LaTeX v0.1

Table 1. Performance of the GCN to mild COVID patients hybrid model. First-appearing figures stand for model 1 while the second ones stand for model 2 with aggregation (see Methods).

	precision	recall	f1-score	support
**False**	0.24/0.50	0.36/0.05	0.29/0.08	22
**True**	0.67/0.72	0.54/0.98	0.60/0.83	54
**accuracy**			0.49/0.71	76
**macro avg**	0.46/0.61	0.45/0.51	0.44/0.46	76
**weighted avg**	0.55/0.65	0.49/0.71	0.51/0.61	76

DRAFT
