# Supplementary material for: Plasma proteome dynamics of COVID-19 severity learnt by a graph convolutional network of multi-scale topology
Source: Life Sci Alliance. 2023 Feb 20;6(5):e202201624. doi: 10.26508/lsa.202201624 (PMC9941303; doi:10.26508/lsa.202201624)
Supplement: Supplementary file 10 [file LSA-2022-01624_TableS2.pdf]

Genome Research draft LaTeX v0.1

Table 2. Performance of the GCN to intermediate COVID patients hybrid model. First-appearing figures stand for model 1 while the second ones stand for model 2 with aggregation (see Methods).

	precision	recall	f1-score	support
**False**	0.14/0.30	0.65/0.15	0.23/0.20	20
**True**	0.87/0.88	0.37/0.94	0.52/0.91	126
**accuracy**			0.41/0.84	146
**macro avg**	0.51/0.59	0.51/0.55	0.38/0.55	146
**weighted avg**	0.77/0.80	0.41/0.84	0.48/0.81	146

DRAFT
